# Synthesis and Antibacterial Activity of Polyoxometalates with Different Structures

**DOI:** 10.1155/2018/9342326

**Published:** 2018-12-09

**Authors:** Jingmin Gu, Lei Zhang, Xiaofeng Yuan, Ya-Guang Chen, Xiuzhu Gao, Dong Li

**Affiliations:** ^1^College of Veterinary Medicine, Jilin University, Changchun 130062, China; ^2^Animal Science and Technology College, Jilin Agricultural University, Changchun 130117, China; ^3^Department of Pediatrics, Affiliated Hospital of the Changchun University of Chinese Medicine, Changchun 130021, China; ^4^Key Laboratory of Polyoxometalates Science of Ministry of Education, Faculty of Chemistry, Northeast Normal University, Changchun 130024, China; ^5^Department of Hepatology, The First Hospital, Jilin University, Changchun 130021, China; ^6^Department of Immunology, College of Basic Medical Sciences, Jilin University, Changchun 130021, China

## Abstract

A new inorganic-organic hybrid compound, [{Cu(phen)_2_}_2_(H_4_W_12_O_40_)], was synthesized, and its crystal structure was determined. The Keggin anion H_4_W_12_O_40_^4−^ was grafted with two coordination units {Cu(phen)_2_}, forming an electrically neutral molecule. The antibacterial activity of several polyoxometalate compounds with different anionic structures including the new compound was studied. The results show that the compound **1** can inhibit the growth of *Enterococcus faecalis* FA2 strains and that antibacterial activity of the polyoxometalate compounds is dependent with component elements of POM but is less relative with the anion structures.

## 1. Instruction

Polyoxometalates (POMs) have been shown to exhibit biological activities *in vitro* as well as *in vivo*, including anticancer and antiviral [1], antibacterial [2, 3], antiprotozoal [4, 5], and antidiabetic activities [6]. In the antibacterial activity study of POMs, Tajima found the enhancement of several beta-lactams antibiotics in antibacterial activity to methicillin-resistant *Staphylococcus aureus* under the synergistic action of polyoxometalates, substituted-type POMs K_7_[PTi_2_W_10_O_40_] 6H_2_O, K_7_[BVW_11_O_40_]7H_2_O, [SiFeW_11_O_40_]^6/5−^ and [SiCoW_11_O_40_]^6−^ and lacunary-type POMs [XW_11_O_39_]^n−^ (X = Si, P) and [XW_9_O_34_]^n−^, and proposed the resistant mechanism [7–12]. Inoue et al. reported the enhancement of antibacterial activity of beta-lactam antibiotics, oxacillin, by polyoxometalates (K_6_[P_2_W_18_18O_62_]·14H_2_O, K_4_[SiMo_12_O_40_]·3H_2_O, and K_7_[PTi_2_W_10_O_40_]·6H_2_O) against methicillin-resistant *Staphylococcus aureus* (MRSA) and vancomycin-resistant *S. aureus* (VRSA) and also proposed a reaction mechanism [13]. Daima et al. studied synergistic antibacterial action of Ag nanoparticles and POMs which were achieved by the physical damage to the bacterial cells [14]. Li and his colleagues showed the short peptides/HSiW nanofibers had antimicrobial activity to the ubiquitous and clinically relevant bacterium *Escherichia coli* [15]. In recent years, there are also many reports about the antibacterial activity of known and new polyoxometalate derivatives to several bacteria including *Escherichia coli*, *Staphylococcus aureus*, *Paenibacillus* sp., *Bacillus subtilis*, *Clavibacter michiganensis*, *Vibrio* sp., *Pseudomonas putida*, *Helicobacter pylori*, *S. typhimurium*, *Streptocoque B* (*S. agalactiae*), *L. acidophilus*, and amebas [3,16–34]. In these reports, the polyoxometalate derivatives are in the form of simple inorganic salts, inorganic-organic hybrids with various organic groups, films, nanofibers, etc. However, the antibacterial activity of these compounds is not satisfactory according to the reported data. Nevertheless, the emergence of multidrug-resistant bacterial strains which was partially due to the abuse of conventional antibiotics proved that there is an urgent need for novel therapeutic agents. Therefore, synthesizing and exploring new compounds with high antibacterial activity are still a challenging task of chemists and pharmacologist. To achieve this, the study on influence of the composition and structure of compounds on antibacterial activity is very important, which will play an instructional role in synthesizing and exploring new compounds.

This work is about the synthesis of a new polyoxometalate derivative, [Cu_2_(phen)_4_(H_4_W_12_O_40_)], and study on the antibacterial activity of several polyoxometalate compounds with different anionic structures including the new compound.

## 2. Materials and Methods

### 2.1. Materials and General Methods

All reagents were purchased commercially and used without further purification. Elemental analyses (C, H, and N) were performed on a Perkin–Elmer 2400 CHN elemental analyzer and that of W and Cu on an ICP–AES analyzer. The IR spectrum was obtained on an Magna-560 FT/IR spectrometer with KBr pellets in the 400–4000 cm^−1^ region. TG analysis was carried out on a DTG-60H thermal analyzer in flowing N_2_ with a heating rate of 10°C·min^−1^. SEM images were recorded on Hitachi S-3400N (Hitachi High-Technologies Europe GmbH, Krefeld, Germany).

### 2.2. Synthesis

Synthesis of [Cu_2_(phen)_4_(H_4_W_12_O_40_)] was modified from our previous report [35]: compound **1** was prepared from reaction of (NH_4_)_6_(H_2_W_12_O_40_)·3H_2_O (0.1 mmol, 0.30 g), CuCl_2_ 2H_2_O (2.0 mmol, 0.34 g), phenanthroline (0.5 mmol, 0.099 g), succinic acid (0.5 mmol, 0.06 g), and 12 mL water. The starting mixture was adjusted to pH = 2.0 by the addition of hydrochloric acid, and the mixture was stirred for 1 h under air. The final solution was transferred to a 25 mL Teflon-lined autoclave and crystallized at 160°C for 96 h. Then, the autoclave was cooled at the rate of 10°C·h^−1^ to room temperature. The resulting green stripe crystals were filtered off, washed with distilled water, and air-dried. Good-quality crystals were sealed for structural determination and further characterization. Elemental analysis calcd for C_48_H_32_Cu_2_N_2_O_41_W_12_ (*Mr* = 3710) C 1.00, H 12.7619, N 2.48, O 20.68, Cu 3.38, P 1.10, W 58.60 (%); found: C 1.10, H 12.39, N 2.41, O 20.56, Cu 3.39, P 1.09, W 59.05 (%). IR(KBr pellet, cm^−1^): 3500, 3082, 2370, 2298, 2109, 1994, 1628, 1597, 1524, 1335, 1231, 1085, 948, 781, 750, 708, 667, 593, 530 cm^−1^.

Compounds **2–6** were prepared in accordance with the methods in Refs. [36–39] and characterized by the IR spectrograph and TGA.

### 2.3. X-Ray Crystallography

The X-ray diffraction data of compound **1** were collected on a Bruker Smart Apex II diffractometer with graphite monochromatic Mo K*α* radiation (*λ* = 0.710 73 Å) at 293 K with *ω* scans (Table 1). Multiscan absorption corrections were applied. The structures were solved by direct methods and refined by full matrix least-squares on *F* ^2^ using the SHELXTL crystallographic software package [40]. The positions of hydrogen atoms on the carbon atoms were calculated theoretically. Crystal data and structure refinements for compound **1** are presented in Table 1. Cu-O and Cu-N bond lengths are listed in Table 2. CCDC-1487664 for **1** contains the supplementary crystallographic data for this paper. These data can be obtained free of charge from The Cambridge Crystallographic Data Centre via http://www.ccdc.cam.ac.uk/data_request/cif.

### 2.4. Antibacterial Experiments

All the isolated bacterial strains were achieved by colony formation on selective salt agar plates containing 6 mg/mL oxacillin. All bacterial strains were stored at −80°C and routinely grown at 37°C. *Staphylococcus aureus* (YB57), *Enterococcus faecalis* (FA2 and FA3), and *Enterococcus faecium* (SA2 and SA3) strains were cultured in brain heart infusion (BHI) broth, while *Staphylococcus aureus* (USA300), *Acinetobacter baumannii* (ABC3), and *Streptococcus pneumoniae* (SP) were cultured in the Luria-Bertani (LB) medium. The polyoxometalates were tested for their antibacterial activities against eight different bacterial strains by the observation of the OD value of culture media. Briefly, bacterial cells were washed and resuspended in sterile PBS, and the colony count was determined. The different polyoxometalates were added to the bacterial suspension (final concentration, 1 mg/mL), and the mixture was incubated overnight at 37°C. The colony count was determined again. Enzyme activity *in vitro* was expressed as the CFU reduction. As a negative control, the bacterial strains were treated with the elution buffer under the same conditions. The results are listed in Table 3.

### 2.5. Scanning Electron Microscopy

Scanning electron microscopy (SEM) was performed to assess the activity of different polyoxometalates on the bacterial strains *in vitro*. The *Staphylococcus aureus* strains USA300 were grown to the exponential growth phase (an OD 600 nm value of 0.6) in the BHI broth at 37°C with shaking at 200 rpm. The bacteria were collected and washed three times (5,000 × g for 1 min at 4°C) with PBS. Different formulations were separately added to *S. aureus* suspensions. Bacterial lysates were harvested by centrifugation (1,100 × g for 1 min) at different time points. Then, the bacterial lysates were fixed with glutaraldehyde and were dehydrated and freeze-dried for SEM.

## 3. Results and Discussion

### 3.1. Crystal Structure of **1**

**Figure 1:**
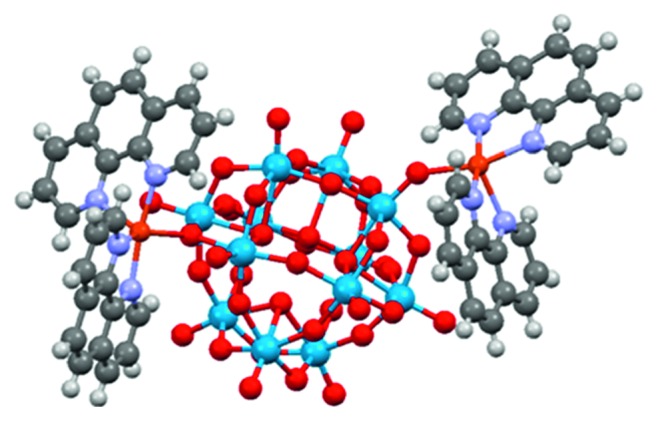
Ball-stick representation of the decorated Keggin anion in **1**.

The asymmetric unit of compound **1** consists of one Keggin anion [H_4_W_12_O_40_]^6−^, two Cu^2+^ ions, and four phen molecules. The [H_4_W_12_O_40_]^6−^ anion (Figure 1) contains four edge-shared W_3_O_13_ units which combine together through corner-shared linkage. W–O bonds can be classified into three sets: W–O_t_ (terminal oxygen atoms) with distances of 1.683(12)–1.740(12) Å, W–O_b_ (bridging oxygen atoms) with distances of 1.847(12)–2.010(14) Å and W–O_c_ (central oxygen atoms) with distances of 2.174(12)–2.396(13) Å. That is, the WO_6_ octahedra are all distorted. The Keggin anion acts as a bidentate ligand bonding two Cu^2+^ ions (Cu1 and Cu2) with one terminal oxygen atom and one bridge oxygen atom. One W-O_t_ bond was elongated (1.740(12) Å) due to the coordination of the terminal oxygen atom to Cu ion. Such a POM anion is also called as decorated Keggin anion (Figure 1), very similar to the decoration we reported previously [35].

Two Cu^2+^ ions are all five-coordinated. Cu1 ion displays in a square prism geometry, and the geometry of Cu2 ion is better to be described as triangle bipyramid. The donor atoms bonding to Cu ions come from two phen molecules with chelating coordination mode and the Keggin anion, forming a complex fragment {Cu(phen)_2_}^2+^ (Figure 1). Cu-O and Cu-N bond lengths are listed in Table 2. As shown in Table 2, the long bonds belong to the atoms at axial site for Cu1 and triangle plane for Cu2 (Figure S1), resulting from their environment in the crystal. Devi et al. [41] had reported a similar cluster [{Cu(phen)2}2(H2W12O40)]^2·̶^ in [{Cu(phen)2}4{H2W12O40}] [{Cu(phen)2}2{H2W12O40}]·3H2O, in which one Cu ion is six-coordinated, different from that of this new compound. The neutral molecules are assembled into three dimensional architecture through CH ⋯ O hydrogen bonds (Table S2) and intermolecular interaction force (Figure S2).

### 3.2. Characterization of **1**

The IR spectrum (Figure S3) of **1** shows the vibration absorption bands of CH bond in 3080 cm^−1^ and of C-C and N-C bonds of phen ring in 1614–1137 cm^−1^. The vibration absorption bands of compound **1** at 952, 877, 846, and 740 cm^−1^ should be ascribed to the asymmetric stretching vibrations of W-O_d_, W-O_b_-W, and W-O_c_-W bonds, respectively, consistent with that in Ref. [42]. The TG curve of **1** is shown in Figure S4. Compound **1** is stable below 400°C and then decomposes until 600°C. The lost weight of 20.17% is consistent with the calculated one (20.38% for 2H_2_O and 4phen), confirming the chemical formula obtained from elemental analysis and structure analysis.

### 3.3. Antibacterial Activity of **1–6**

The compounds **1–6** used in antibacterial experiments can be divided into four kinds. **1** and **2** are inorganic-organic hybrids with phenanthroline, and **3** is a mono-substituted Keggin-type compound in which Ti atom occupies one of twelve sites. **4** is a complex of mono-lacunaria Lindquist anion and lanthanides. **5** and **6** are complexes of mono-lacunaria Keggin anions and lanthanides. From Table 3, it can be seen that (1) the new compound **1** is active only to bacterial strains FA2. (2) The compound of molybdenum, **2**, has a wider antibacterial spectrum than that of tungsten (**1**, **3**, **4**, **5**, **6**). (3) The anionic structure has less influence on antibacterial activity. (4) The compounds with cerium element (**4**, **6**) show better antibacterial activity than others.

**Figure 2:**
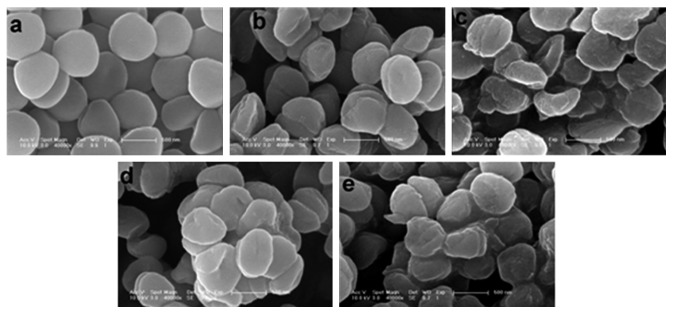
SEM images of *Staphylococcus aureus* strains USA300 untreated (a) and treated with **3** (b), **4** (c), **5** (d), and **6** (e).

SEM technique was used to explore the interaction of polyoxometalates with the bacterial strains. SEM images (Figure 2) show the surface morphology of *Staphylococcus aureus* strains USA300 untreated (Figure 2) and treated with **3**, **4**, **5**, and **6** (Figures 2–2). From Figure 2, it can be seen that the surface morphology of *Staphylococcus aureus* strains USA300 treated with polyoxometalates (Figures 2–2) has changed obviously compared with that of untreated one (Figure 2) from smooth globular form to chapping oblate spheroid. The degree of changes in the surface morphology indicates the antibacterial activity of polyoxometalates. So, a sequence of the activity of polyoxometalates was given according to Figure 2, **4** ≈ **6** > **5** > **3**. That is, the compounds with cerium element (**4**, **6**) have better antibacterial activity than others.

## 4. Conclusion

The bioactivity of polyoxometalates has been known for many years but still has large space to explore. The results of this work on the antibacterial activity of polyoxometalates including the new compound show that antibacterial activity of the compounds is more relative with their component element than with anionic structure, which means that exploration of antibacterial materials should focus on the choice of elements.

In this work, the compounds with Ce elements have better antibacterial activity. So, synthesizing compounds with other lanthanide elements and other compounds with cerium element and examining their antibacterial activity as well as exploring the reaction mechanism of Ce compounds need further investigations.

## Figures and Tables

**Table 1:** Crystal data and structure refinements for compound **1**.

Formula	C_48_H_32_Cu_2_N_8_O_40_W_12_
Fw	3710
Crystal system	Monoclinic
Space group	P21/c
*a*/Ǻ	26.1828(15)
*b*/Ǻ	11.84219(7)
*c*/Ǻ	23.3996(13)
*α*/˚	90.00
*β*/˚	113.74(2)
*γ*/˚	90.00
*V*/Ǻ^3^	6641.2(7)
*Z*	4
*D*_c_/g·cm^−3^	3.711
F(000)	6600
*μ*/mm^−1^	21.419
R_int_	0.0932
Refine number of reflns/parameters/restraints	13661/991/54
R_factor_all/[*I* > 2sigma(*I*)]	0.0871/0.0456
wR_factor_ref/[*I* > 2sigma(*I*)]	0.0857/0.0745
Goodness of fit	0.954

**Table 2:** Cu-O and Cu-N bond lengths (Å) in **1**.

Bond	Length	Bond	Length
Cu1 N1	2.214(11)	Cu2 N5	1.960(12)
Cu1 N2	1.990(10)	Cu2 N9	2.038(12)
Cu1 N3	1.996(13)	Cu2 N7	1.965(12)
Cu1 N4	1.991(11)	Cu2 N8	2.168(12)
Cu1 O7	2.045(10)	Cu2 O38	2.217(8)

**Table 3:** Antibacterial activities of compounds **1–6**.

Type	Code	Compound	Concentration	Bacterial strains
I	1	[{Cu(phen)_2_}_2_(H_4_W_12_O_40_)]	1 mg/mL	FA2
	2	[Cu(phen)(H_2_O)(Mo_3_O_10_)]	1 mg/mL	ABC3, FA2, FA3, YB57, USA300
II	3	K_5_PW_11_TiO_40_·14H_2_O	1 mg/mL	YB57, USA300
III	4	Na_7_CeW_10_O_35_·26H_2_O	1 mg/mL	SA5, SA1, SP, USA300
IV	5	K_13_[La(SiW_11_O_39_)_2_]·26H_2_O	1 mg/mL	YB57, USA300
	6	K_13_[Ce(SiW_11_O_39_)_2_]·26H_2_O	1 mg/mL	SA5, SA1, SP, USA300

## Data Availability

The data used to support the findings of this study are included within the article.

## Supplementary Materials

Supplementary Materials Figure S1: coordination geometries of Cu ions; Figure S2: stacking representation of **1** from three directions of (A) *a*-axis, (B) *b*-axis, and (C) *c*-axis; Figure S3: IR spectrum of **1**; Figure S4: TG plot of **1** in 0‐600°C; Table S1: W-O bond lengths (Å); Table S2: CH···O hydrogen bonds in **1**.
