# Supplementary material for: Synthesis and Antibacterial Activity of Polyoxometalates with Different Structures
Source: Bioinorg Chem Appl. 2018 Dec 9;2018:9342326. doi: 10.1155/2018/9342326 (PMC6305049; doi:10.1155/2018/9342326)
Supplement: Supplementary Materials — Figure S1: coordination geometries of Cu ions; Figure S2: stacking representation of 1 from three directions of (A) a-axis, (B) b-axis, and (C) c-axis; Figure S3: IR spectrum of 1; Figure S4: TG plot of 1 in 0‐600°C; Table S1: W-O bond lengths (Å); Table S2: CH···O hydrogen bonds in 1. [file 9342326.f1.pdf]

Supporting information

Synthesis and antibacterial activity of polyoxometalates with different structures

Jingmin Gu [1#], Lei Zhang [2#], Xiaofeng Yuan [3], Ya-Guang Chen [4], Xiuzhu Gao [5], Dong Li [5, 6 *]

Table S1 W-O bond lengths (Å)

Bond	Length	Bond	Length	Bond	Length	Bond	Length
W1 O34	1.722(8)	W2 O32	1.698(9)	W3 O17	1.710(9)	W4 O30	1.712(9)
W1 O13	1.847(9)	W2 O1	1.865(10)	W3 O23	1.896(8)	W4 O19	1.911(8)
W1 O14	1.917(9)	W2 O10	1.893(8)	W3 O12	1.921(10)	W4 O21	1.915(8)
W1 O11	1.936(10)	W2 O2	1.942(10)	W3 O6	1.945(9)	W4 O11	1.920(9)
W1 O38	2.010(8)	W2 O27	2.007(8)	W3 O38	1.949(8)	W4 O12	1.933(9)
W1 O35	2.210(8)	W2 O24	2.284(8)	W3 O35	2.271(8)	W4 O35	2.392(9)
W5 O7	1.740(10)	W6 O33	1.704(10)	W7 O37	1.683(8)	W8 O8	1.709(9)
W5 O25	1.883(8)	W6 O19	1.892(8)	W7 O6	1.869(8)	W8 O3	1.883(9)
W5 O2	1.896(8)	W6 O15	1.898(9)	W7 O18	1.880(10)	W8 O16	1.901(9)
W5 O21	1.906(8)	W6 O26	1.906(8)	W7 O15	1.939(10)	W8 O1	1.938(10)
W5 O22	1.956(9)	W6 O25	1.942(8)	W7 O29	2.077(8)	W8 O39	1.959(9)
W5 O24	2.221(9)	W6 O40	2.396(9)	W7 O40	2.226(8)	W8 O20	2.299(8)
W9 O4	1.698(10)	W10 O31	1.710(9)	W11 O36	1.709(9)	W12 O5	1.709(8)
W9 O9	1.915(8)	W10 O39	1.891(8)	W11 O22	1.888(11)	W12 O27	1.807(9)
W9 O18	1.934(9)	W10 O28	1.907(10)	W11 O14	1.893(8)	W12 O16	1.902(9)
W9 O23	1.934(8)	W10 O9	1.932(9)	W11 O28	1.913(10)	W12 O26	1.933(9)
W9 O3	1.951(8)	W10 O13	1.966(8)	W11 O10	1.950(8)	W12 O29	2.112(8)
W9 O20	2.207(9)	W10 O20	2.338(8)	W11 O24	2.311(8)	W12 O40	2.174(7)

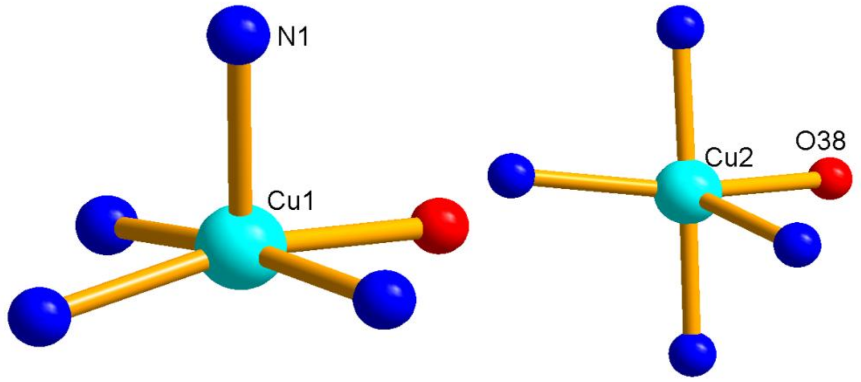

FigS1 Coordination geometries of Cu ions

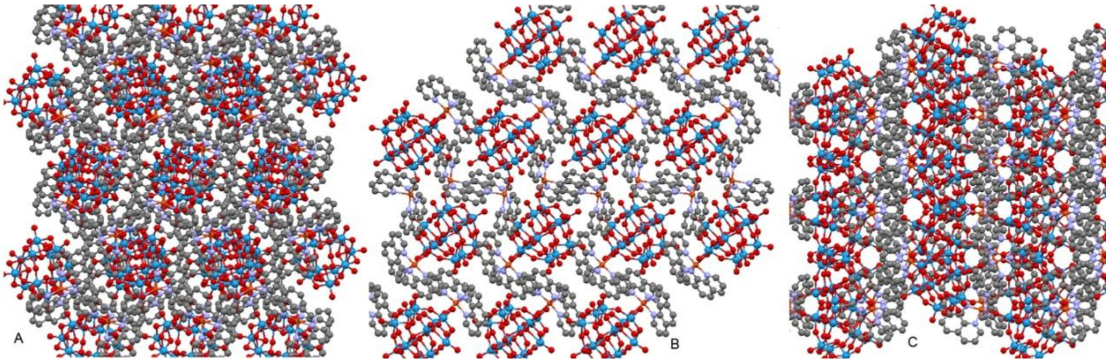

Fig S2 Stacking representation of **1** from three directions of (A) *a*-axis, (B) *b*-axis and (C) *c*-axis

Table S2 CH···O hydrogen bonds in **1**

D-H	*d*(D-H)	*d*(H..A)	<DHA	*d*(D..A)	A
C10-H10A	0.930	2.335	154.31	3.199	O39 [x, -y+3/2, z-1/2]
C22-H22A	0.930	2.325	145.04	3.132	O4 [-x+1, -y+2, -z+1]
C22-H22A	0.930	2.512	130.61	3.196	O9 [-x+1, -y+2, -z+1]
C23-H23A	0.930	2.571	110.75	3.028	O7

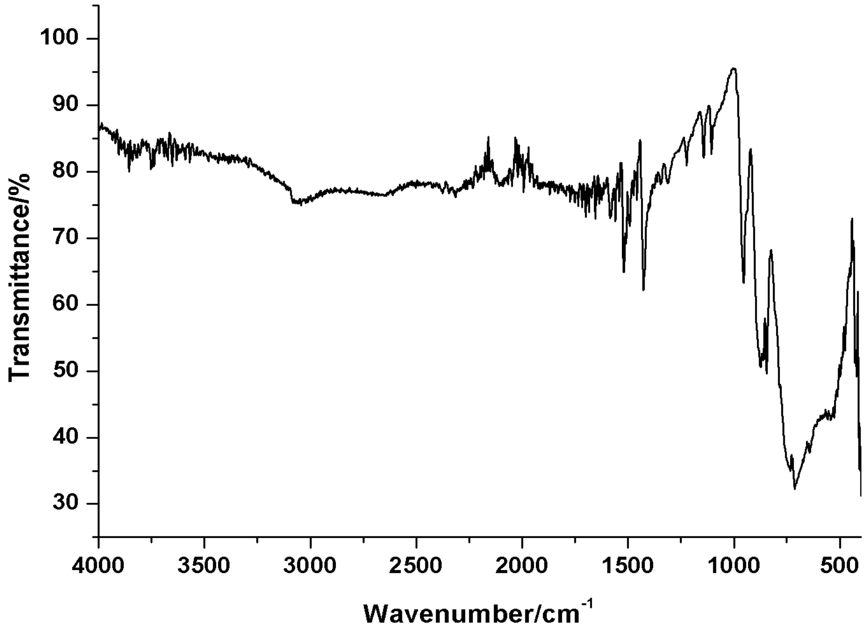

Fig S3 IR spectrum of **1**

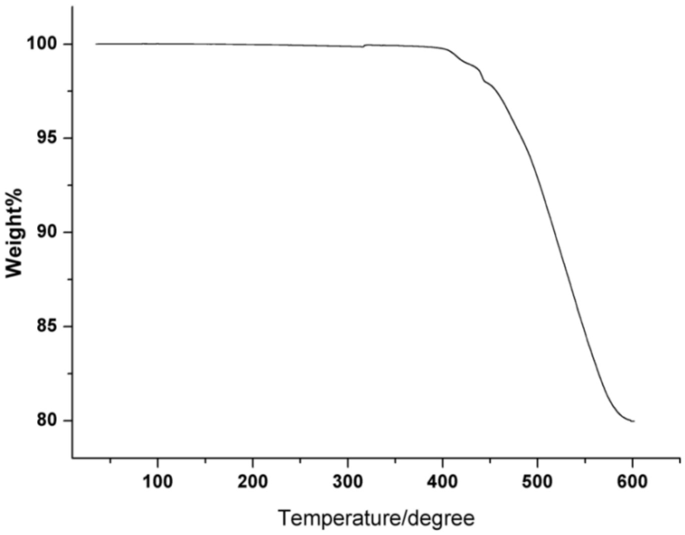

Fig S4 TG plot of **1** in 0-600 ℃
